# Complete Chloroplast Genome Sequence of Cane Needle Grass, *Nassella hyalina* (Poaceae: Stipeae)

**DOI:** 10.1128/genomeA.01215-17

**Published:** 2017-11-22

**Authors:** Aisuo Wang, Hanwen Wu, David Gopurenko

**Affiliations:** NSW Department of Primary Industries, Wagga Wagga Agricultural Institute, PMB, Wagga Wagga, NSW, Australia, and Graham Centre for Agricultural Innovation, Wagga Wagga, NSW, Australia

## Abstract

*Nassella hyalina* (cane needle grass) is on the Alert List for Environmental Weeds in Australia. We present here the first complete chloroplast sequence of *N. hyalina* reconstructed from Illumina whole-genome sequencing. The complete chloroplast sequence is 137,606 bp in size and has a gene content and structure similar to those of other published chloroplast genomes of Stipeae.

## GENOME ANNOUNCEMENT

*Nassella hyalina* (Nees) Barkworth (Poaceae: Stipeae), commonly known as cane needle grass, is on the Alert List for Environmental Weeds in Australia (1). As one of the South American stipoid grasses that are naturalized in southeastern Australia, it poses a significant threat to Australia’s ecosystems (1). The development of genetic diagnostics to distinguish *N. hyalina* from similar-appearing congeners and grasses endemic to Australia, such as *Austrostipa* species, is essential for the effective management of this weed. However, the lack of genomic data, such as the chloroplast genome sequence of this weed, has hindered the development of genetic diagnostic technologies. Here, we sequenced the complete chloroplast genome of *N. hyalina* (collected from Wagga Wagga, NSW, Australia) using Illumina sequencing technology. The specimen was taxonomically identified as *N. hyalina* and retained at the Wagga Wagga Agricultural Institute (WWAI) under voucher number ww19870.

A modified cetyltrimethylammonium bromide (CTAB) protocol (2) was applied to extract total genomic DNA from fresh leaves of the specimen. DNAs that met the quality control (QC) requirements were used for library construction (500-bp insert size) before being sequenced on an Illumina HiSeq 2000 sequencer to generate 125-bp paired-end reads (BGI, Hong Kong). NOVOPlasty (3), an algorithm specifically developed for the *de novo* assembly of mitochondrial and chloroplast genomes from whole-genome data, was applied to extract chloroplast genome sequences from the raw reads of the FASTQ files. The recommended k-mer value (39) was applied, and the chloroplast genome of the pooid grass *Brachyelytrum aristosum* (GenBank accession number NC_027470) was used as a seed input in the analysis. The final assembly resulted in a complete circular genome sequence with a length of 137,606 bp and G+C content of 38.81%.

Annotation of the *N. hyalina* chloroplast genome was performed using both CpGAVAS (4) and DOGMA (5), with default settings, to predict protein-coding genes, tRNA genes, and rRNA genes. The predicted annotations were verified using a BLAST search (6).

The chloroplast genome structure of *N. hyalina* was highly similar to that of other Stipeae chloroplast genomes (such as *Stipa hymenoides* [GenBank accession number NC_027464], *Stipa lipskyi* [NC_028444], *Stipa purpurea* [NC_029390], *Oryzopsis asperifolia* [NC_027479], and *Piptochaetium avenaceum* [NC_027483]). It consisted of two inverted regions (IRs) (21,267 bp), a large single copy (LSC) (81,547 bp), and a small single copy (SSC) (13,541 bp). Of the 106 unique genes (78 protein-coding genes, 4 rRNAs, and 24 tRNAs), 78 genes were located in the LSC region (61 protein-coding genes and 17 tRNA genes), 11 genes were located in the SSC region (10 protein-coding genes and 1 tRNA gene), and 19 genes were located in both IR regions (9 coding genes, 4 rRNA genes, and 6 tRNA genes). A single intron was contained in coding genes *rpoC2*, *atpF*, *rpl2*, *ndhB*, *ndhA*, *ndhB*, and *rpl2*, while double introns were present in *ycf3*.

The availability of the chloroplast genome of *N. hyalina* provides a platform by which further genetic studies (such as DNA barcoding, loop-mediated isothermal amplification [LAMP], etc.) can be conducted, which is useful for the integrated management of this weed.

### Accession number(s).

The complete sequence of the *Nassella hyalina* chloroplast genome was deposited in GenBank under accession number MF480753.
